# Whole-Exome Sequencing Identifies the VHL Mutation (c.262T > C, p.Try88Arg) in Non-Obstructive Azoospermia-Associated Cystic Renal Cell Carcinoma

**DOI:** 10.3390/curroncol29040192

**Published:** 2022-03-28

**Authors:** Yonghong Man, Xuejun Shang, Chunyu Liu, Wei Zhang, Qian Huang, Suheng Ma, Rita Shiang, Feng Zhang, Ling Zhang, Zhibing Zhang

**Affiliations:** 1 Department of Occupational Health and Environmental Medicine, School of Public Health, Wuhan University of Science and Technology, Wuhan 430065, China; manyonghong@wust.edu.cn (Y.M.); 15671620132@163.com (Q.H.); masuheng2810109825@163.com (S.M.); 2 Department of Urology, Medical School of Nanjing University Affiliated Jinling Hospital, Nanjing 210002, China; shangxj98@163.com (X.S.); 18851188851@163.com (W.Z.); 3 Obstetrics and Gynecology Hospital, Fudan University, Shanghai 200011, China; 17111250002@fudan.edu.cn (C.L.); zhangfeng@fudan.edu (F.Z.); 4 Department of Physiology, School of Medicine, Wayne State University, Detroit, MI 48210, USA; 5 Department of Human and Molecular Genetics, Virginia Commonwealth University, Richmond, WI 23298, USA; rita.shiang@vcuhealth.org; 6 Department of Obstetrics & Gynecology, The C.S. Mott Center for Human Growth and Development, Detroit, MI 48201, USA

**Keywords:** renal cell carcinoma, von Hippel-Lindau syndrome, non-obstructive azoospermia, VHL, pVHL, microtubule, cilia, flagellum

## Abstract

Von Hippel-Lindau (VHL) genes are intimately involved in renal cell carcinoma (RCC), including clear cell RCC (ccRCC) pathogenesis. However, the contribution of pathogenic VHL mutations to ccRCC remains poorly understood. We report a xanthoderm with non-obstructive azoospermia (NOA)-associated cystic ccRCC, and the missense VHL mutation (c.262T > C, p.Try88Arg). In a 34-year-old patient, a urologic physical examination identified hard epididymis, and imaging tests revealed deferens-associated NOA, as well as multi-organ hydatid cysts, including bilateral epididymal cysts, bilateral testicular cysts, bilateral renal cysts, and pancreatic cysts. Five years later, ccRCC was developed based on clinical and radiologic evidence. Two different prediction models of protein structure and multiple sequence alignment across species were applied to assess the pathological effects of the VHL mutation. The reliability of the assessment in silico was determined by both the cellular location and protein levels of the mutant products, using IF and Western blot, respectively. Our study shows that the missense VHL mutation (c.262T > C, p.Try88Arg) plays a deleterious role in pVHL functions, as predicted by multiple sequence alignment across species. While a structural analysis identified no significant structural alterations in pVHL, the detrimental effects of this mutation were determined by exogenous expression, evidenced by a markedly different spatial distribution and reduced expression of mutant pVHL. This is the first report of the VHL gene mutation (c.475T > C, p.Try88Arg) in a xanthoderm.

## 1. Introduction

Renal cell carcinoma (RCC) is a common genitourinary cancer, accounting for approximately 2~3% of adult neoplasms. Worldwide, there are an estimated 270,000 new cases of RCC and 116,000 deaths due to kidney cancer yearly [1]. In the United States, there were about 63,000 new cases and almost 14,000 deaths due to RCC in 2016; in fact, RCC has become the ninth most prevalent cancer in America [2]. Four major RCC entities have been identified, including clear cell renal cell carcinoma (ccRCC) [3], papillary renal cell carcinoma (pRCC) [4], chromophobe renal cell carcinoma (chrRCC) [5], and collecting duct carcinoma (CDC) [6]. Among these four histological subtypes, ccRCC is the predominant subtype of renal cancer, and accounts for the majority of kidney cancer deaths [7]. Autosomal dominantly inherited pathogenic germline variants have been implicated in the pathogenesis of ccRCC, such as in the VHL, c-Met, BaP1, and Pbrm1 genes [8,9,10], in which pathogenic variants in VHL have been identified as the leading cause of ccRCC pathogenesis [11].

In mammals, pVHL is a target recruitment subunit in the E3 ubiquitin ligase complex VCB–CR. Binding to partner proteins, including Elongin B (elob), Elongin C (eloc), Cullin 2 (cul2), and rbx1, pVHL delivers protein substrates to the VCB–CR complex for ubiquitination [12]. The substrates of the VCB–CR complex include hypoxia-inductive factors (HIFs) [13]. Moreover, dysregulation of the VHL–HIF axis has been shown to induce oncogenic cellular processes and contribute to ccRCC pathogenesis [14].

NOA is heterogeneous in etiology, and genetic problems, such as chromosomal abnormalities and gene mutations, are important causes [15]. The VHL, which is an autosomal dominant disorder, resulting from deletion or mutation in the VHL gene, has been linked to obstructive azoospermia [16]. This heritable cancer syndrome increases a person’s risk of developing benign and malignant lesions in multiple organ systems, including ccRCC [17]. However, no specific VHL gene mutation has been reported in ccRCC secondary to NOA, and the precise nature of the VHL gene mutation in the pathogenesis of NOA and ccRCC remains unclear.

Here, we identified a heterozygous missense mutation in the VHL gene from a patient with azoospermia and multiple-organ cystic renal cell carcinoma. Comprehensive clinical evaluation included imaging findings and pathological studies. In silicon analyses, including online mutation prediction software and multiple sequence alignment across species, were performed to predict the pathological effects of the VHL mutation (c.262T > C, p.Try88Arg). The single amino substitution, however, exerted no significant alteration to the 3D structure of pVHL, as predicted by the server Missense3D. Nevertheless, an ectopic expression of the mutant gene in vitro showed a remarkable change in the distribution pattern and expression level of mutant pVHL. As the mutational spectra of VHL have been identified, there is great interest in studying the correlation between the VHL gene mutation and its clinical signature, especially proband symptoms. Our data provide a necessary incentive to understand the role of VHL in NOA and ccRCC pathogenesis. They also suggest that a missense mutation can be functionally meaningful in the absence of a commensurate change in the predicted protein structure, and, therefore, it may be missed by a single structural analysis of a protein. Finally, genetic analysis of VHL, combined with andrological diagnosis, could be necessary for the early diagnosis and prognosis of ccRCC.

## 2. Materials and Methods

### 2.1. Patient and Clinical Evaluation

The patient presented here was a 34-year-old xanthoderm male, visiting a comprehensive hospital with clinical complaints of NOA and urinary tract discomfort, which were diagnosed previously. Bilateral epididymis and Vas deferens were checked by physical exams. The epididymis and testis were checked using a B-ultrasound examination. Enhanced CT plain scan of the abdominal cavity and magnetic resonance imaging (MRI) of the pelvic cavity were performed to identify the nature and extent of multiple-organ cysts.

### 2.2. Isolation of Genomic DNA and Whole-Exome Sequencing

Total genomic DNA was extracted from the 200 μL whole blood of the patient and his parents using the QIAamp DNA blood mini kit (Qiagen, Hilden, Germany) according to the manufacturer’s protocol. About 5 µg of purified DNA was diluted in 200 µL of buffer AE. Whole-exome sequencing was performed on a HiSeq X ten PE150 by Novogene Co., Ltd. (Shanghai, China). The resulting data were analyzed and annotated using the DNAnexus (https://www.dnanexus.com/ 20 March 2020) data storage and analysis facility.

### 2.3. Sanger Verification and Restriction Enzyme Digestion Site Analysis

The presence of potential variants was confirmed by PCR and Sanger sequencing using standard protocols. The region containing the mutant polymorphism was amplified by polymerase chain reaction (PCR) using the following primer set: 5′-TGAAGAAGACGGCGGGGAG-3′ and 5′-CTCGGTAGCTGTGGATGCGGCG-3′. The reactions were denatured at 95 °C for 3 min, followed by 35 cycles of 95 °C for 30 s, 52 °C for 1 min and 72 °C for 1 min. The expected size of the PCR products for VHL is 266 bp, which contains nucleotide 262. Given that a T262C substitution creates an Rsa Ⅰ recognition sequence, the product was digested using the Rsa Ⅰ restriction enzyme. The digested PCR products were separated by electrophoresis using 8% agarose gel in the presence of ethidium bromide.

### 2.4. Vector Construction, Site-Directed Mutagenesis, Cellular Localization, and Expression Analyses

VHL cDNA was amplified from human-VHL (NM_000551.4)-cDNA-pDONR223 (Youbio, Changsha, China) and cloned into pEGFP-N2 with the following primer set: 5′-GAATTCATGCCCCGGAGGGCGGAGAACTG’ (forward), 5′-GGATCCCATCTCCCATCCGTTGATGTGCA-3′ (reverse), to create a vector called pEGFP-N2-VHL (WT). Successful ligation of VHL cDNA with pEGFP-N2, and verification that the inserted sequence was free of mutation, was confirmed by complete nucleotide sequencing (Sangon-Biotech, Shanghai, China). The mutation (NM_000551.4:c.262T > C) was introduced into pEGFP-N2-VHL (WT) by amplifying the entire pEGFP-N2-VHL vector with the following primer set: 5′-GCCCGTATGGCTCAACTTCGACGGC-3′ (sense), 5′-GCCGTCGAAGTTGAGCCATACGGGC-3′ (antisense). The resulting PCR products were then digested with Dpn1 at 37 °C for 1 h and directly transformed into E. coli DH5α. The pEGFP-N2-VHL (p.Try88Arg) clones were sequenced to confirm the mutation.

For pVHL localization studies, Lipofectamine™ 2000 (Invitrogen, Waltham, MA, USA) was used to transfect HEK293T with 100 ng of pEGFP-N2-VHL (WT) or pEGF-N2-VHL (p.Try88Arg) according to the manufacturer’s instructions. Following overnight incubation, cells were washed with ice-cold PBS and mounted with DakoCytomation fluorescence mounting media. Images were acquired using a Zeiss LSM 510 Meta confocal microscope (Carl Zeiss, AG, Jena, Germany) with a 63× oil differential interference contrast objective.

To compare the protein expression levels of wild-type VHL with the mutant VHL, the two plasmids were transfected into COS-1 cells. Forty-eight hours after transfection, cells were collected with RIPA buffer, and Western blot was conducted with an anti-GFP antibody. The level of GAPDH was also examined to normalize the VHL levels.

## 3. Results

### 3.1. Clinical Findings

**Figure 1:**
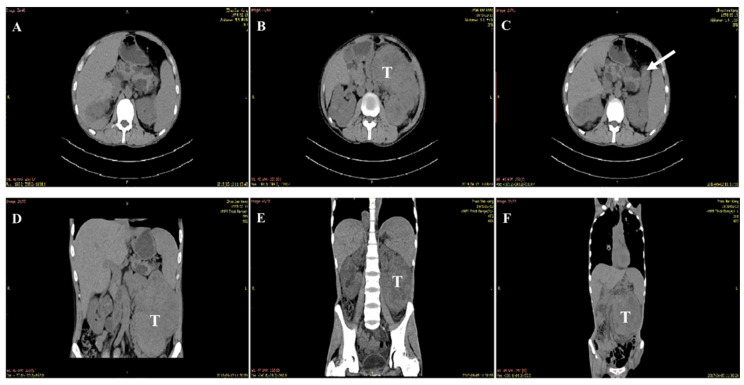
Pathological findings. (**A**) This image shows a normal transverse CT scan. (**B**) This transverse CT scan shows a large mass lesion with low-density cystic lesions (T), arising from the left kidney. (**C**) This transverse CT scan shows an aberrant extension of the left kidney (white arrow). (**D**–**F**) Coronal CT scans show that the RCC originated from the left kidney and they also provide information regarding tumor size.

The physical exams detected the hard texture of bilateral epididymis, and the bilateral vas deferens were thin and stiff. B ultrasound identified multiple cysts in both the epididymis and testis. To determine the nature and extent of the cyst’s lesion, enhanced CT plain scans of the abdominal cavity and magnetic resonance imaging (MRI) of the pelvic cavity were performed. Multi-organ hydatid cysts, including bilateral epididymal cysts, bilateral testicular cysts, bilateral renal cysts, and pancreatic cysts, were revealed. The patient was diagnosed with polycystic kidney, with epididymal cysts. Five years after his diagnosis, the patient’s symptoms progressively worsened, and he eventually died of ccRCC, based on clinical and radiologic evidence (Figure 1).

### 3.2. Identification of a Missense Mutation in VHL

**Figure 2:**
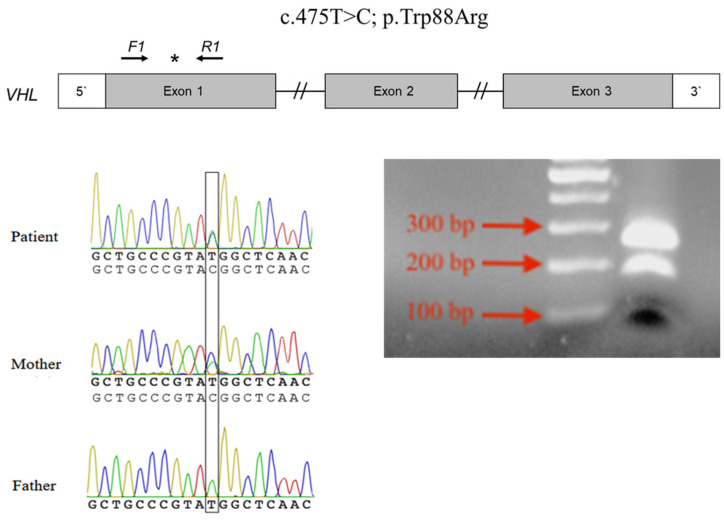
Detection of VHL gene mutation (c.262T > C) in the patient and his family. (* refers to the mutant site in the amplicon).

The VHL coding sequence is represented in three exons (Figure 2). The heterozygous missense mutation (NM_000551.4: c.262T > C) in exon 1 of the VHL gene was identified in the patient and his mother by whole-exome sequencing. Sanger sequencing confirmed that the patient and his mother were carrying this mutation in a heterozygous form (Figure 2). His father was devoid of this mutation. This mutation was further detected using the PCR-RFLP method. Digestion of the 266-bp amplicon resulted in either retention of the 266-bp product or complete digestion to 187-bp and 79-bp fragments (Figure 2).

The human VHL cDNA (NM_000551.4) was organized exonically. The star indicates the mutation location, and the arrows indicate the region of the PCR primers. Figure 2 illustrates partial DNA sequences in the VHL gene by Sanger sequencing of the patient and his parents. Figure 2 shows that the amplicon (266-bp) of the 262T mutant allele contains an Rsa I cleavage site, and was digested into 187-bp and 79-bp fragments.

### 3.3. An Overall Analysis of the VHL p.Trp88Arg Mutation

**Figure 3:**
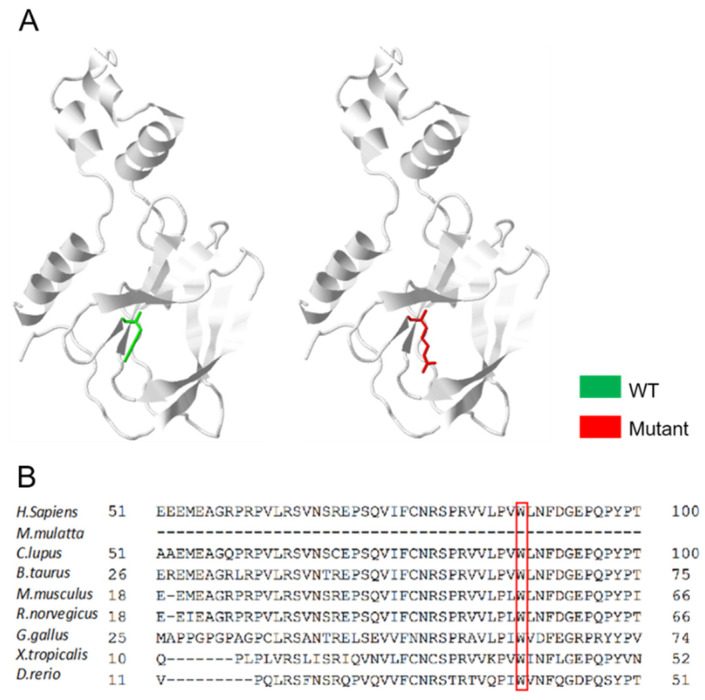
In silico analysis of pVHL. (**A**) Missense3D does not predict a structural change introduced by a TRP (wild type) > ARG (mutant) substitution; (**B**) multiple sequence alignment of the mutant residue (p.Trp88Arg) in wild-type VHL protein of several species. (The red recktangle refers to the conserved amino among different species).

The missense mutation (NM_000551.4: c.262T > C) was not identified in the genome aggregation database (gnomAD v2.1.1, https://gnomad.broadinstitute.org/ 15 November 2021). However, it is present in the dbSNP database (dbSNP: rs1553619431), UniProtKB database (code VAR_005697) and ClinVar database (https://www.ncbi.nlm.nih.gov/clinvar/ 15 November 2021), without its distribution frequency. This missense mutation leads to the replacement of tryptophan with arginine at the 88th position (p.Try88Arg) in the VHL-encoded protein and VHL tumor suppressor protein (pVHL). The predicted 3D structures of proteins provide reliable insights into whether missense variants are associated with disease, while it is also important to assess the reliability of using predicted models when analyzing missense variants [18]. Interestingly, no structural change in pVHL was predicted for p.Trp88Arg using Missense3D (http://www.sbg.bio.ic.ac.uk/missense3d/ 11 November 2021) (Figure 3A). Therefore, another in silico analysis was performed to understand and predict the significance of this mutation. Online mutation prediction software, PolyPhen-2 and I-Mutant 2.0, predicted this mutation as “PROBABLY DAMAGING” and “Deleterious”, respectively. In the multiple sequence alignment, the VHL p.Trp88Arg is evolutionarily highly conserved among different species (Figure 3B). Hence, we predict that the mutation of this residue may exert a dominant negative effect on pVHL.

### 3.4. Mutation Changed Localization of pVHL and Reduced Its Protein Level in Transfected Mammalian Cells

**Figure 4:**
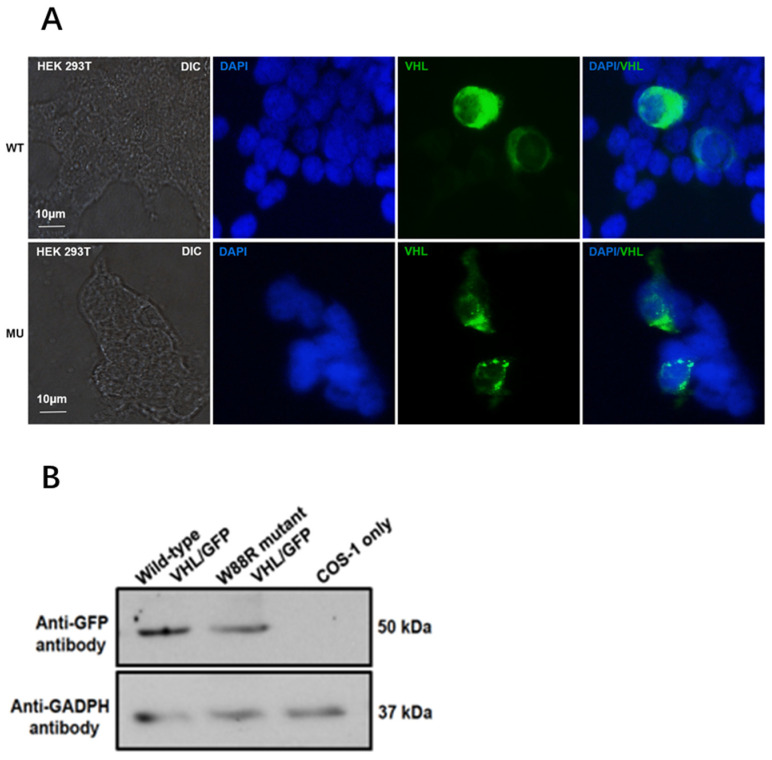
VHL mutation (p.Try88Arg) changes the intracellular localization and protein level. (**A**). Confocal microscopy shows the intracellular localization of GFP-pVHL (WT) and GFP-pVHL (p.Try88Arg) in HEK293 cells. The targeted plasmids were transfected into HEK293 for 24 h, and the cells were counterstained with DAPI. The cells were then washed three times and used for imaging. GFP-pVHL (green); DAPI (blue). (**B**). Western blot analysis showed a reduced VHL protein level in HEK293 cells transfected with the VHL mutant expression vector.

To examine whether the mutation (NM_000551.4: c.262T > C) affected pVHL expression and localization in the cells, we constructed a GFP-fused VHL wild type, pEGFP-N2-VHL (WT). Using site-directed mutagenesis, we constructed a GFP-fused VHL mutant expression vector, pEGFP-N2-VHL (p.Try88Arg). We compared the cellular localization of pVHL (WT) and pVHL (p.Try88Arg) in HEK293 cells, and found that the wild-type VHL-GFP protein was uniformly expressed in the cytoplasm. However, the mutant VHL-GFP protein aggregated into granules in the cytoplasm (Figure 4A). These results indicate that this VHL mutation changes the localization of the protein in the cell.

The VHL levels were also examined in the transfected cells, by Western blot analysis. Compared to the wild-type VHL, the mutant VHL level was dramatically reduced (Figure 4B).

## 4. Discussion

In this study, we identified a VHL gene mutation in a 34-year-old male patient exhibiting NOA-related cystic ccRCC. Whole-exome sequencing revealed a missense mutation in the first exon of the VHL gene. Although multi-species alignment analysis confirmed the conservation of this amino acid, Missense3D predicted that the mutation exerts no effect on the biophysical structure and proteostasis of pVHL. Remarkably, the exogenous expression of the VHL gene in cells revealed significant changes in both the distribution pattern of pVHL within the cytoplasm and the protein levels.

The discovery of the VHL gene stemmed from VHL syndrome (von Hippel-Lindau syndrome), which is an autosomal dominant hereditary disease [19]. This syndrome is related to the occurrence of a variety of angiogenic tumors, including ccRCC. Moreover, 56% to 91% of patients suffering from sporadic ccRCC have been identified with mutant alleles of the VHL gene [18]. These mutations can affect the overall proteostasis or specific functional properties of pVHL, due to the thermodynamic instability of proteins [7]. In this study, the deleterious consequences of the mutation on the pVHL structure were not established by protein 3D structural mutation prediction, but the risk was predicted by PolyPhen-2 and I-Mutant 2.0 analysis. In this case, analysis-based evolutionary conservation provided a more reliable tool for predicting the potential disease severity of gene mutations. Although evolutionary stress reduces the prevalence of high-risk missense mutations in the population, it provides clues for understanding the biology of ccRCC.

pVHL has the function of stabilizing microtubules, which are the main components of cilia axoneme. Cilia are hairy organelles that protrude from most types of cell surfaces. The axoneme of a cilium is an axial filament based on microtubules, with nine peripheral doublet microtubules and a pair of central microtubules (9 + 2 structure) [20]. Flagella, also cellular surface organelles, share the same core structure (axial filament) as cilia. Disorders of sperm flagella formation and motility are linked to asthenospermia, which plays a deleterious role in male infertility [21]. Notably, the patient in our study also presented with an onset symptom of reproductive disorders. The significance of this manifestation in surveillance strategies for ccRCC should be further verified in a large-scale examination.

Cilia play an essential role in the development of, and normal physiological activities of, tissues and organs [22]. Defects of ciliary assembly or ciliary signal transduction disorders are involved in various human disease pathogeneses, termed ciliopathies. These include breast cancer, prostate cancer, lung cancer [23], medulloblastoma [24], renal cell carcinoma, and renal cysts, which are considered to be a precursor of ccRCC [23]. Our study also found that the male patient developed systemic multiple-organ cysts, dominated by renal cysts.

Notably, the VHL gene is closely related to cilia formation and function. Its functional protein pVHL maintains the stability of the structure and function of cilia by mediating ubiquitin degradation and regulating the level of Hypoxia-inducible factor 1-alpha (HIF-1α) [25]. It is worth noting that pVHL inactivation can induce the expression of HEF1/Cas-L/NEDD9 and AuroraA proteins by stabilizing HIF-1. This activates histone deacetylase 6 (HDAC6), which depolymerizes the microtubules of cilia axoneme and causes cilia reabsorption/degeneration [26]. Moreover, pVHL inactivation can also up-regulate the expression of the HIF-α downstream genes AIX and Nek8. The expression products of AIX and Nek8 participate in the occurrence of polycystic kidney and renal cell carcinoma, by affecting cilia function [27,28,29]. In this study, the exogenic expression of mutant pVHL was shown to modify the cellular distribution pattern compared with its wild-type counterpart, which may also affect cilia-related signaling pathways.

It has been reported that VHL-deficient mice display typical VHL syndrome, and a remarkable phenotype was impaired spermatogenesis [30]. However, it is unclear what percentage of male VHL patients are afflicted with male factor infertility. In this case, the original chief complaint of the patient was infertility. The patient’s symptoms progressively worsened, and ccRCC developed 5 years after diagnosis. This alternation might suggest a role of infertility in the prelude of ccRCC, particularly with a VHL gene mutation. Thus, follow-up examinations might be valuable in the early diagnosis of fetal diseases.

## 5. Conclusions

Collectively, we found a new association between the VHL gene mutation and clinical phenotype, which may be used for the early diagnosis and prognosis of VHL-related ccRCC. Our data also indicated that the structural approach in silicon might not be an accurate indicator of the pathological effects of a missense mutation. In addition, the heterogeneity of the VHL mutations helps in the study of the NOA pathogenesis. Future research should explore the contribution of the VHL gene mutation to ccRCC pathogenesis at the cellular and animal levels. The evidence described in this study also highlights the importance of considering cilia dysfunction as a possible mechanism underlying ccRCC.

## Data Availability

All data generated or analyzed during this study are included in this article. Further inquiries can be directed to the corresponding author.
